# The association between Acute Lymphoblastic Leukemia in children and *Helicobacter pylori* as the marker for sanitation

**DOI:** 10.1186/1756-0500-5-345

**Published:** 2012-07-03

**Authors:** Pengiran Hishamuddin

**Affiliations:** 1 Surveillance and Response Branch, Communicable Diseases Division, Ministry of Health, 16 College Road, Singapore, 169854, Republic of Singapore

**Keywords:** Public health, Epidemiology, Oncology

## Abstract

**Background:**

Greaves “delayed infection” hypothesis suggested that Acute Lymphoblastic Leukemia (ALL) in children is caused by a lack of exposure to infection in infancy, which may be due higher standards of sanitation. We have conducted an ecologic analysis of the relationship between sanitation, using *Helicobacter pylori (H. pylori)* as the marker, and the incidence of childhood ALL in 127 cancer registries from 28 countries.

**Results:**

There were inverse associations between *H. pylori* prevalence and ALL incidence rates in children. These associations were minor and only significant for ALL incidence rates for all cancer registries. They became non-significant and smaller in magnitude when the population source and/or the GNP per capita were added to the relationship. Furthermore, these results were unchanged when the associations were examined using the Generalized Estimating Equations.

**Conclusions:**

Although the findings showed lower prevalence of *H. pylori* and improved sanitation is associated with increased incidence of childhood ALL, they do not conclusively support Greaves “delayed infection” hypothesis.

## Background

Leukemia is the most common cancer in children [1]. The major types of childhood leukemia are acute lymphoblastic leukemia (ALL) and acute myeloid leukemia (AML) [1,2]. ALL accounts for the majority of childhood leukemia [1,2]. The incidence of childhood ALL has increased over the past 20 years [1,2]. This trend is more apparent among 2 to 5 years and in developed countries [1-3].

Genetic alterations occurring during fetal development increase the risk of childhood ALL [4,5]. However, these genetic factors are insufficient for leukemogenesis, which appears to require a “second hit” [6]. One potential mechanisms of such “second hit” transition is proposed by the “delayed infection” (or Greaves) hypothesis [7]. According to the Greaves’ hypothesis, ALL in children is caused by a lack of exposure to infections in infancy. This inadequate exposure to infections in infancy leads to failure of modulation in the immune system, which involves changes to certain T-cells [7]. Exposure to one or more common bacterial or viral infections later in life, for example after mixing with carriers such as children in playgroups or schools [8,9], then results in an abnormal immune response and eventually ALL. This delayed infection may be due to improved sanitary conditions found in modern lifestyles [7]. Greaves’ “delayed infection” hypothesis is conceptually similar to the “hygiene hypothesis” that has been proposed to explain allergies, asthma and type 1 diabetes [10]. The “hygiene hypothesis” suggests that early childhood infections are needed to promote the development of the immune system and to suppress allergic and autoimmune disorders.

This study examines Greaves’ “delayed infection” hypothesis by looking at whether level of sanitation is associated with the incidence rates of childhood ALL in different countries. In this study we use *Helicobacter pylori (H. pylori)* as a marker of sanitation. *H. pylori* is an ubiquitous bacterium affecting a large percentage of the world’s population [11]. Prevalence of *H. pylori* is greater in people who reside in conditions with inadequate saniation [12]. Therefore, lower prevalence of *H. pylori* is associated with improved sanitation and lack of exposure to infections. Under the “delayed infection” hypothesis countries with lower prevalence of *H. pylori* are expected to have a higher incidence rate of childhood ALL and vice versa.

## Methods

### ALL incidence rates

The ALL incidence rates (per 100, 000 persons) in 0 to 4 years old children obtained from the monograph, Cancer Incidence in Five Continents, Volume VIII, published jointly by the International Agency for Research on Cancer (IARC) and the International Association of Cancer Registries (IARC) [13]. Data were available from 185 cancer registries in 56 countries. However, the analyses were confined to those countries in which corresponding *H. pylori* prevalence rates for children were available. These comprised 28 countries that include 127 cancer registries. All the data were collected between 1991 and 1998, with the majority (78%) collected between 1993 and 1997.

### Weighted ALL incidence rates

Weighted ALL incidence rates, which is the pooled estimate of the ALL incidence rates for cancer registries in a country, were calculated using the inverse variance method where larger studies are given more weight than smaller studies [14]. If I_i_, (i = 1,2 …), is the ALL incidence rate for the i^th^ cancer registry in a country, then the weighted ALL incidence rate for the entire country is defined as **(∑ I**_**i**_**w**_**i**_**) / (∑ w**_**i**_**)**, where the registry-specific weight (w_i_) equals 1/(s_i_^2^) and s_i_^2^ represents the variance of the I_i_ estimate. As ALL incidence rates are binomially distributed, the variance (s_i_^2^) for the ALL incidence rate for each individual cancer registry was obtained using the formula **s**^2^_**i**_ **= n**_**i**_**I**_**i**_**(1-I**_**i**_**),** where n_i_ (i = 1, 2 …) is the population size of children aged 0 to 4 years old in the i^th^ cancer registry, and I_i_ (i = 1, 2 …) is the ALL incidence rate for the i^th^ cancer registry in a country [15].

### *H. pylori* prevalence rates

The prevalence rates (per 100 persons) of *H. pylori* infection in children from 26 countries were obtained from studies reviewed by Torres et al [16]. To supplement these data, a Medline search of articles using the search term *Helicobacter pylori* and prevalence was conducted and *H. pylori* prevalence rates for two additional countries, Singapore [17] and Thailand [18], were found.

Only one *H. pylori* prevalence rate, for children between 0 to 10 years old was used for each country. The prevalence rates/studies included in the analyses were selected based on the following criteria:

Studies which looked exclusively at infants aged 0 to 1 years old were excluded, as the *H. pylori* prevalence in this age group, especially when measured using the serological assays, could be influenced by maternal IgG antibodies that were transferred to the child [16].

The maximum age of children in the studies was limited to 10 years old as the most relevant group for ALL children is under this age [1-3].

Studies with children 1 to 4 years old were preferred, and if this was not available then the next youngest age group was selected.

### Covariates

Certain characteristics of the *H. pylori* studies were considered as potential confounders. All *H. pylori* surveys were characterized as either clinic-based or population-based. Clinic-based surveys can be affected by selection bias because participants in these studies may have higher socioeconomic status and live in better sanitary conditions [16]. As a result *H. pylori* prevalence rates are more likely to be lower in clinic-based samples. Another potential confounder was the method of *H. pylori* detection. The studies used either the urea breath test or serological assays of IgG antibodies. The *H. pylori* prevalence rates differ depending on which method was utilized, and this is due to the different sensitivities and specificities associated with these diagnostic tests [19]. The degree of urbanization in the area where the studies were conducted was also a potential confounder as *H. pylori* prevalence rates have been shown to be higher in more urban areas [16]. The minimum and maximum ages of children in the studies, were considered potential confounders as *H. pylori* prevalence rates and ALL incidence rates are known to change according to age groups [16]. *H. pylori* prevalence rates also vary according to geographic locations [16,19], and therefore the regional location of a country was considered a potential confounder. To examine this, all countries that provided the data for analyses were categorized according to the World Health Organization (WHO) regions [20]. The data for each country also included the interval between the year of the ALL incidence and the year when the *H. pylori* survey was conducted.

Characteristics of the countries considered in this study included the Gross National Product (GNP) per capita, Human Development Index (HDI) and the population size. GNP per capita is defined as the dollar value of a country’s final output of goods and services in a year, divided by its population size [21]. This reflects the average income of a country’s citizens. HDI measures a country’s average achievements in human development [22]. It consists of three components – life expectancy at birth, education levels, and standards of living as measured by the Gross Domestic Product per capita. GNP per capita and HDI are considered to be markers for the level of affluence and development of a country, and also potential confounders for *H. pylori* prevalence and ALL incidence rates. This is because countries with higher GNP per capita and HDI typically have improved levels of sanitation and healthcare, and are likely to have lower *H. pylori* prevalence and higher ALL incidence rates. For each country, the analytic dataset included the GNP per capita and the population size corresponding to the year the *H. pylori* study [21]. The United Nations Development Program releases HDI data every 5 years [22]. The specific HDI value was selected based in its proximity to the *H. pylori* survey.

### Statistical analyses

The ALL incidence rates were considered using two methods: the weighted method combined the data from all cancer registries is each country (n = 28) and the non-weighted method included data from each cancer registry separately (n = 127). The *H. pylori* prevalence rate was continuously entered into all the models.

The minimum and maximum age of children in the *H. pylori* studies, the interval between the ALL incidence and *H. pylori* surveys, the GNP per capita, the HDI, and the population size were treated as continuous variables. The method of *H. pylori* detection (urea breath test versus serological assay), the population source (clinic-based versus community-based), the level of urbanization (urban, rural or both) and the WHO region (Africa, Americas, South East Asia, Europe and Western Pacific) were treated as categorical variables.

All analyses were performed using the software SAS 9.1.3.

#### Univariate analyses

PROC UNIVARIATE was used to look at the normal distributions, mean, median, and standard deviation of all continuous variables. PROC FREQ was used to produce counts and cross tabulation tables for all categorical variables. Both the continuous and categorical variables were examined for missing values and extreme outliers.

Both outcome variables (the weighted ALL incidence rates for each country and the ALL incidence rates for all cancer registries) were normally distributed. Only one other continuous covariate, the interval between ALL incidence and *H. pylori* surveys, was normally distributed. No issues with regards to missing values were found in all the continuous and categorical variables. No problems with extreme outliers were identified in continuous variables.

#### Bivariate analyses

All bivariate analyses were performed twice, initially with the weighted ALL incidence rate for each country (n = 28) and then repeated with the ALL incidence rate for all cancer registries (n = 127) in the model. PROC CORR was used to perform correlations between:

Both outcome variables and the *H. pylori* prevalence rates.

Both outcome variables and the other continuous covariates.

The *H. pylori* prevalence rates and the other continuous covariates.

The other continuous covariates with each other.

The r^2^ value, which denotes the percentage of variation in the dependent outcome variable which is accounted for by the independent exposure variable or covariates, and the significance of the correlation (p-value of less than 0.05) was looked at. Pearson’s method was used to look at the correlation between both outcome variables and the interval between ALL incidence and *H. pylori* surveys. This method was applied as both the outcome variables and this continuous covariate were normally distributed. Spearman’s method was used for all other correlations as at least one continuous variable then was not normally distributed. For categorical variables, PROC GLM or PROC ANOVA was performed to look at the mean, standard deviation and significance (p-value of less than 0.05) of the prevalence of both outcome variables, *H. pylori* prevalence rate, the other continuous and categorical covariates, in each category of the categorical covariates.

#### Bivariate analyses: the correlations between ALL incidence, *H. pylori* prevalence and characteristics of *H. pylori* studies/countries (n = 28)

The correlations between all continuous variables when the weighted ALL incidence rates for each country (n = 28) were modeled were calculated using PROC CORR. Pearson’s method was used for the correlation between the weighted ALL incidence rates for each country and the interval between the ALL incidence and the *H. pylori* surveys, as both these variables were normally distributed. Spearman’s method was used for all other correlations as at least one continuous variable then was not normally distributed. The weighted ALL incidence rates for each country was negatively correlated to the *H. pylori* prevalence rates but this was not significant (r^2^ = −0.14, p-value = 0.48). The covariates which were not significantly correlated with both the weighted ALL incidence rates for each country and the *H. pylori* prevalence rates were the minimum and maximum age of children in the *H. pylori* studies, the interval between the ALL incidence and the *H. pylori* surveys and the population size of the countries. These 4 covariates could potentially be excluded from the model. Significant correlations identifying potential confounders were:

*H. pylori* prevalence rates and GNP per capita (r^2^ = −0.60, p-value = 0.0007).

*H. pylori* prevalence rates and HDI (r^2^ = −0.57, p-value = 0.002).

Significant correlation identifying potential collinearity was:

GNP per capita and HDI (r^2^ = 0.95, p-value = <0.0001)

#### Bivariate analyses: the correlations between ALL incidence, *H. pylori* prevalence and characteristics of *H. pylori* studies/countries (n = 127)

The correlations between all continuous variables when the ALL incidence rates for all cancer registries in all countries (n = 127) were modeled were calculated using PROC CORR. Pearson’s method was used for the correlations between the ALL incidence rates for all cancer registries in all countries and the interval between the ALL incidence and the *H. pylori* surveys, as both these variables were normally distributed. Spearman’s method was used for all other correlations as at least one continuous variable then was not normally distributed. The ALL incidence rates for all cancer registries in all countries was negatively correlated to the *H. pylori* prevalence rates but this was not significant (r^2^ = −0.17, p-value = 0.05). The covariates which were not significantly correlated with both the ALL incidence rates for all cancer registries and the *H. pylori* prevalence rates were the maximum age of children in the *H. pylori* studies and the interval between the ALL incidence and the *H. pylori* surveys. These 2 covariates could potentially be excluded from the model.

Significant correlations identifying potential confounders were:

ALL incidence rates for all cancer registries and GNP per capita (r^2^ = 0.38, p-value = <0.0001)

ALL incidence rates for all cancer registries and HDI (r^2^ = 0.41, p-value = <0.0001)

ALL incidence rates for all cancer registries and population size of countries (r^2^ = −0.21, p-value = 0.019).

*H. pylori* prevalence rates and GNP per capita (r^2^ = −0.43, p-value = <0.0001).

*H. pylori* prevalence rates and the HDI (r^2^ = −0.40, p-value = <0.0001).

*H. pylori* prevalence rates and the population size of countries (r^2^ = 0.54, p-value = <0.0001).

*H. pylori* prevalence rates and the minimum age of children in the *H. pylori* studies (r^2^ = 0.24, p-value = 0.003).

Significant correlations identifying potential collinearity were:

GNP per capita and the HDI (r2 = 0.97, p-value = <0.0001).

GNP per capita and population size (r2 = −0.25, p-value = 0.005).

HDI and population size of countries (r2 = −0.26, p-value = 0.003).

#### Bivariate analyses: the relation of ALL incidence, *H. pylori* prevalence, the minimum and maximum age of children, the interval between ALL incidence and *H. pylori* surveys, GNP per capita, HDI and population size to various characteristics of *H. pylori* studies (n = 28)

PROC GLM was performed to look at the mean, standard deviation and significance (p-value of less than 0.05) of the prevalence of the weighted ALL incidence rates for each country, the *H. pylori* prevalence rates and the other continuous covariates, in each category of the categorical covariates (which were certain characteristics of the *H. pylori* studies). The prevalence of both the weighted ALL incidence rates for each country and the *H. pylori* prevalence rates was not significant in the method of *H. pylori* used in the *H. pylori* studies. This covariate could potentially be excluded from the model.

Significant associations identifying potential confounders were:

The prevalence of *H. pylori* prevalence rates in the population source used in the *H. pylori* survey (p = 0.04), the level of urbanization in the *H. pylori* studies (p = 0.02) and the region a country is situated (p = 0.0003).

Significant associations identifying potential collinearity were:

The prevalence of GNP per capita in the level of urbanization in the *H. pylori* studies (p = 0.02).

The prevalence of HDI in the level of urbanization in the *H. pylori* studies (p = 0.008) and the region a country is situated (p = <0.0001).

#### Bivariate analyses: the relation of ALL incidence, *H. pylori* prevalence, the minimum and maximum age of children, the interval between ALL incidence and *H. pylori* surveys, GNP per capita, HDI and population size to various characteristics of *H. pylori* studies (n = 127)

PROC GLM were performed to look at the mean, standard deviation and significance (p-value of less than 0.05) of the prevalence of the ALL incidence rates for all cancer registries, the *H. pylori* prevalence rates and the other continuous covariates, in each category of the categorical covariates (which were certain characteristics of the *H. pylori* studies). The prevalence of both the ALL incidence rates for all cancer registries and the *H. pylori* prevalence rates was not significant in the method of *H. pylori* detection used in the *H. pylori* studies. This covariate could potentially be excluded from the model.

Significant associations identifying potential confounders were:

The prevalence of the ALL incidence rates for all cancer registries in the population source used in the *H. pylori* survey (p = 0.038), the level of urbanization in the *H. pylori* studies (p = 0.002) and the region a country is situated (p = 0.0008).

The prevalence of *H. pylori* prevalence rates in the population source used in the *H. pylori* survey (p = <0.0001), the level of urbanization in the *H. pylori* studies (p = <0.0001) and the region a country is situated (p = <0.0001).

Significant associations identifying potential collinearity were:

The prevalence of GNP per capita in the level of urbanization in the *H. pylori* studies (p = <0.0001) and the region a country is situated (p = <0.0001).

The prevalence of HDI in the population source used in the *H. pylori* survey (p = 0.004), the level of urbanization in the *H. pylori* studies (p = <0.0001) and the region a country is situated (p = <0.0001).

The prevalence of the population size of countries in the population source used in the *H. pylori* survey (p = 0.0006), the level of urbanization in the *H. pylori* studies (p = <0.0001) and the region a country is situated (p = <0.0001).

#### Bivariate analyses: the associations among various characteristics of *H. pylori* studies (n = 28)

PROC ANOVA were performed to look at the mean, standard deviation and significance (p-value of less than o.05) of the prevalence of the categorical covariates in each other, when the weighted ALL incidence rates in each country was modeled.

Significant associations identifying potential collinearity was:

The prevalence of the population source used in the *H. pylori* survey in the level of urbanization in the *H. pylori* studies (p = 0.007).

#### Bivariate analyses: the associations among various characteristics of *H. pylori* studies (n = 127)

PROC ANOVA were performed to look at the mean, standard deviation and significance (p-value of less than 0.05) of the prevalence of the categorical covariates in each other, when the ALL incidence rates in all cancer registries were modeled.

Significant associations identifying potential collinearity were:

The population source used in the *H. pylori* survey in the level of urbanization in the *H. pylori* studies (p = <0.0001).

The level of urbanization in the *H. pylori* studies in the population source used in the *H. pylori* survey (p = 0.002) and the region a country is situated (p = <0.0001).

#### Covariates or confounders excluded from further analyses

Covariates which were not correlated or associated with either *H. pylori* prevalence of ALL incidence rates were not included in the model. The confounders that remained were examined for collinearity. When this existed one confounder was chosen to be included in the model based on the strength of its correlation or association to either *H. pylori* prevalence or ALL incidence rates.

#### Continuous covariates or confounders excluded from further analyses

GNP per capita and the HDI were positively correlated with ALL incidence rates. This correlation was not significant when weighted ALL incidence rates were modeled (GNP per capita: r^2^ = 0.22, p-value = 0.26; HDI: r^2^ = 0.18, p-value = 0.36) but was significant when the ALL incidence rates for all cancer registries were modeled (GNP per capita: r^2^ = 0.38, p-value = <0.0001; HDI: r^2^ = 0.41, p-value = <0.0001). GNP per capita and the HDI were negatively correlated with the *H. pylori* prevalence rates. For example, when the ALL incidence rates for all cancer registries were used as the outcome variable, the correlation between *H. pylori* prevalence rates and GNP per capita had a r^2^ of −0.43 and a p-value of <0.0001 whereas the correlation between *H. pylori* prevalence rates and HDI had a r^2^ of −0.40 and a p-value of <0.0001. GNP per capita and HDI were highly correlated to each other. For example, when the weighted ALL incidence rates for each country were used in the model, the correlation between these two was significantly positive with a r^2^ of 0.95 and a p-value of <0.0001. This meant that both covariates could not be considered in the same model as doing so might lead to potential collinearity. GNP per capita was the covariate used in the further analyses based on its higher correlation to the *H. pylori* prevalence and ALL incidence rates.

The population size of the countries was negatively correlated with the ALL incidence rates. This correlation was significant when the ALL incidence rates for all cancer registries were modeled (r^2^ = −0.21, p-value = 0.019). The population size of countries was positively correlated with the *H. pylori* prevalence rates. This correlation was significant when the outcome variable was the ALL incidence rates in all cancer registries (r^2^ = 0.54, p-value = <0.0001). The population size of a country was negatively correlated with a confounder, GNP per capita. This correlation was significant when the ALL incidence rates for all cancer registries were modeled (r^2^ = −0.25, p-value = 0.005). This is not surprising as the denominator in the calculation of the GNP per capita of a country is its population size. The population size of a country was therefore excluded from further analysis.

The minimum age of children in the *H. pylori* studies was positively correlated with the *H. pylori* prevalence rates. This correlation was significant when the outcome variable was the ALL incidence rates for all cancer registries (r^2^ = 0.24, p-value = 0.003). This covariate was not consistently correlated with both ALL incidence rates. When the weighted ALL incidence rates for each country were modeled, the correlation had a r^2^ of 0.34 with a p-value of 0.08 and when the ALL incidence rates for all cancer registries were modeled, the correlation had a r^2^ of −0.008 with a p-value of 0.93. Therefore, the minimum age of children in the *H. pylori* studies was excluded from further analysis.

The maximum age of children in the *H. pylori* studies was not consistently correlated with both ALL incidence rates. When the weighted ALL incidence rates for each country were modeled, the correlation had a r2 of −0.23 with a p-value of 0.24 and when the ALL incidence rates for all cancer registries were modeled, the correlation had a r2 of 0.10 with a p-value of 0.29. This covariate was also not consistently correlated with the *H. pylori* prevalence rates. When the weighted ALL incidence rates for each country were modeled, the correlation with the *H. pylori* prevalence rates had a r^2^ of 0.24 with a p-value of 0.21 and when the ALL incidence rates for all cancer registries were modeled, the correlation with the *H. pylori* prevalence rates had a r^2^ of −0.08 with a p-value of 0.38. Therefore, the maximum age of children in the *H. pylori* studies was not used in the further analysis.

The interval between the ALL incidence and the *H. pylori* surveys was not consistently correlated with both ALL incidence rates. When the weighted ALL incidence rates for each country were modeled, the correlation had a r^2^ of 0.21 with a p-value of 0.27 and when the ALL incidence rates for all cancer registries were modeled, the correlations had a r2 of −0.15 with a p-value of 0.08. The interval between the ALL incidence and the *H. pylori* surveys was not consistently correlated with the *H. pylori* prevalence rates. When the weighted ALL incidence rates for each country were modeled, the correlation with the *H. pylori* prevalence rates had a r^2^ of −0.01 with a p-value of 0.94 and when the outcome variable was the ALL incidence rates for all cancer registries, the correlation with the *H. pylori* prevalence rates had a r^2^ of −0.009 with a p-value of 0.92. Therefore the interval between the ALL incidence and the *H. pylori* surveys was not used in the further analysis. This meant that the only continuous confounder in the model was GNP per capita.

#### Categorical covariates or confounders excluded from further analyses

The method *of H. pylori* detection used in the *H. pylori* studies was not associated with *H. pylori* prevalence for both ALL incidence rates. For example when the weighted ALL incident rates for each country were modeled, the association between the method of *H. pylori* detection used in the *H. pylori* studies and the *H. pylori* prevalence rates had a p-value of 0.14 and between the method of *H. pylori* detection used in the *H. pylori* studies and ALL incidence rates had a p-value of 0.08. This covariate was therefore excluded from further analysis.

The population source used in the *H. pylori* survey was associated with ALL incidence rates for all cancer registries (p-value = 0.038). The type of population source used in the *H. pylori* survey was also associated *H. pylori* prevalence rates for both outcomes. For example, when the ALL incidence rates for all cancer registries were modeled, the associations between the population source used in the *H. pylori* survey and *H. pylori* prevalence rates had a p-value of <0.0001.

The level of urbanization in the *H. pylori* studies was associated with ALL incidence rates for all cancer registries (p-value = 0.002). The level of urbanization in the *H. pylori* studies was also associated with *H. pylori* prevalence rates for both outcomes. For example, when the ALL incidence rate for all cancer registries were modeled, the association between level of urbanization in the *H. pylori* studies and the *H. pylori* prevalence rates had a p-value of <0.0001.

The region each country was situated in was associated with ALL incidence rates for all cancer registries (p-value = 0.0008). The region each country was situated in was also associated *H. pylori* prevalence rates for both outcomes. For example, when the ALL incidence rates for all cancer registries were modeled, the association between region each country was situated in and *H. pylori* prevalence rates had a p-value of <0.0001.

The level of urbanization in the *H. pylori* studies and the region each country was situated in were associated with GNP per capita. For example when the ALL incidence rates for all cancer registries were modeled, the association between GNP per capita and the level of urbanization in the *H. pylori* studies and between GNP per capita and the region in each country was situated both had a p-value of <0.0001. The level of urbanization in the *H. pylori* studies and the region each country was situated in were excluded from further analyses as GNP per capita had a higher correlation to the *H. pylori* prevalence and ALL incidence rates.

#### Confounders included in further analyses

Therefore, confounders that remained and which did not have collinearity with each other, were GNP per capita (continuous) and the population source used in the *H. pylori* survey (clinic-based or community-based samples). These two confounders were included in the final analyses.

#### Linear regression – unadjusted and adjusted analyses

The population source used in the *H. pylori* survey was analyzed using a new categorical covariate (*population1*) which was created using dummy variables, where the reference group is community-based population samples. PROC REG was used to perform unadjusted linear regression analyses between the outcome variables and the *H. pylori* prevalence rates, and the confounders – the GNP per capita and the population source used in the *H. pylori* survey. PROC REG was then repeated to perform adjusted linear regression analyses between the outcome variables and the *H. pylori* prevalence rates, controlling for both confounders separately, and then simultaneously. For each step of the analyses the parameter estimate (coefficient), standard error and significance (p-value of less than 0.05) was looked at. All linear regression analyses were performed twice, initially with the weighted ALL incidence rates for each country (n = 28) as the outcome variable and then repeated with the ALL incidence rates for all the cancer registries (n = 127).

#### Generalized Estimating Equations

Generalized Estimating Equations (GEE) [14], were used to assess the relationship between *H. pylori* prevalence and ALL incidence rates among the 127 cancer registries in the 28 countries, accounting for the dependency and repeated observations within countries. PROC GENMOD was used to perform unadjusted analyses between the outcome variable (which in this method is the ALL incidence rates in all cancer registries belonging to the same country) and the *H. pylori* prevalence rates, and the confounders – the GNP per capita and the population source used in the *H. pylori* survey. PROC GENMOD was then repeated to perform adjusted analyses between the outcome variable and the *H. pylori* prevalence rates, controlling for both confounders separately and then simultaneously. For each step of the analyses the parameter estimate (coefficient), standard error and significance (p-value of less than 0.05) was looked at.

#### Regression diagnostics

The assumptions (that there is a linear relationship between the outcome and predictor variables and that the errors are independent and normally distributed with a mean of 0 and σ^2^) of the linear regression models were examined by plotting:

The values of the outcome variable against its predicted values. This plot should look like a 45° line when the X and Y axes are scaled the same.

The studentized residuals against the predicted values for the outcome variable. If the assumption for the constant variance of errors is true then this plot should show a random scatter about 0 and the width of the scatter should be the same.

The studentized residuals against the values of the predictor variables. If the assumption for the constant variance of errors is true then this plot should show a random scatter about 0 and the width of the scatter should be the same. This plot was done for each predictor variable in the model.

Normal probability plot of errors. This checks the normality assumption.

In addition, issues with regards to influential outliers and collinearity were addressed. The assumptions for the linear regression models were met and there were no issues with regards to influential outliers and collinearity.

### **Ethical approval**

As this study was an ecological study which utilized previously published data and which was not research involving human subjects or animals, it required no submission to IRB. This was determined in consultation with the Communicable Diseases Division, Ministry of Health, Singapore.

## Results and discussion

The characteristics of the *H. pylori* studies are shown in Table 1. These together with characteristics of countries included in the analyses are described further in Table 2. Weighted ALL incidence rates for each country (n = 28) had a median of 16.40 per 100, 000 persons (standard deviation = 11.75) as compared to the ALL incidence rates for all cancer registries ( n = 127) which had a median of 21.25 per 100, 000 persons (standard deviation = 12.62). As for the *H. pylori* prevalence (%), this ranged from 3% to 74% and had a median of 19.30%. The children included in the *H. pylori* studies, had a median minimum age of 1.50 years (standard deviation = 2.25 years), and a median maximum age of 5.00 years (standard deviation = 2.43 years). There were more studies using serological assays (n = 25) than urea breath test (n = 3) as the method of *H. pylori* detection. Out of the twenty-eight *H. pylori* studies, eighteen were clinic-based and ten were community-based surveys. Four studies were conducted in urban areas, and only eight studies were done in both settings. The average interval between the ALL incidence and the *H. pylori* surveys was 4.1 years. Half of the studies were conducted in Europe (n = 14), four studies were from the Americas (14.3%) and six were from Western Pacific (21.4%). Only two studies were from Africa (7.1%) and another two studies were from South-East Asia (7.1%). The average GNP per capita for the countries was US$11, 375.79, while the average HDI for the countries was 0.80. In addition it was noted that both the weighted ALL incidence rates for each country (n = 28) and the ALL incidence rates for all cancer registries (n = 127) were normally distributed.

**Table 1:** **Characteristics of *H. pylori *studies **

**Country**	**H. pylori prevalence**^**a**^	**Min. age**^**b**^	**Max. age**^**c**^	**No.**^**d**^	**Method**^**f**^	**Population source**^**g**^	**Level of urbanization**^**h**^	**Year**^**i**^	**Region**^**k**^	**Reference**
Algeria	45	0	9	42	Serology	Clinic	Urban	1987	Africa	Megraud et al. [23]
Brazil	36.7	3	5	60	Serology	Clinic	Urban	1992	Americas	Oliveira et al. [24]
Colombia	61	2	5	373	UBT	Community	Rural	1992	Americas	Goodman et al. [25]
Costa Rica	60	7	10	60	Serology	Community	Rural	1984-1988	Americas	Sierra et al. [26]
USA	35	1	5	182	Serology	Clinic	Both	1998	Americas	Elitsur et al. [27]
China	53	3	4	19	UBT	Community	Rural	1994	Western Pacific	Ma et al. [28]
India	6	3	10	30	Serology	Clinic	Urban	1989	Southeast Asia	Graham et al. [29]
Japan	12.5	1	4	56	Serology	Clinic	Urban	1994	Western Pacific	Matsukura et al. [30]
Korea	6	1	3	52	Serology	Clinic	Both	1998^j^	Western Pacific	Malaty et al. [31]
Vietnam	13	0	9	61	Serology	Clinic	Urban	1987	Western Pacific	Megraud et al. [23]
Belgium	6.4	1	5	n/a^e^	Serology	Clinic	Both	1995 ^j^	Europe	Blecker et al. [32]
Estonia	4.9	9	9	94	Serology	Community	Both	1993-1996	Europe	Vorobja et al. [33]
Finland	6	3	3	131	Serology	Community	Both	1980	Europe	Ashorn et al. [34]
France	4	0	9	113	Serology	Clinic	Urban	1987	Europe	Megraud et al. [23]
Germany	8.3	3	5	36	Serology	Clinic	Urban	1984	Europe	Hornemann et al. [35]
Iceland	9	0	9	13	Serology	Clinic	Both	1991-1992	Europe	Bergenzaum et al. [36]
Italy	22	1	10	32	Serology	Clinic	Urban	1995	Europe	Luzza et al. [37]
Netherlands	9	6	8	80	Serology	Clinic	Urban	1993	Europe	Roosendaal et al. [38]
Poland	16.6	3	5	60	Serology	Clinic	Urban	1996	Europe	Czkwianianc et al. [39]
Portugal	42.6	3	6	n/a^e^	Serology	Clinic	Urban	1994 ^j^	Europe	Quina et al. [40]
Russia	30	1	4	44	Serology	Community	Urban	1996	Europe	Malaty et.al [41]
Spain	11	2	9	203	Serology	Clinic	Both	1991-1992	Europe	Cilla et al. [42]
Sweden	7.7	1	4	661	Serology	Clinic	Urban	1984-1995	Europe	Grandstorm et al. [43]
Switzerland	7	5	7	432	UBT	Community	Both	1998	Europe	Boltshauser et al. [44]
Australia	25	2	4	20	Serology	Clinic	Urban	1991	Western Pacific	Hardikar et al. [45]
Gambia	31	0	5	353	Serology	Community	Rural	1990	Africa	Sullivan et al. [46]
Singapore	3	0	4	305	Serology	Community	Urban	1992	Western Pacific	[17]
Thailand	74	1	4	27	Serology	Community	Urban	1990	Southeast Asia	Perez-Perez et al. [18]

^a^ H. pylori prevalence (%).

^b^ Minimum age of children in the studies.

^c^ Maximum age of children in the studies.

^d^ Number of children in the age group.

^e^ Only the prevalence rate was available.

^f^ Method of detection was either by serological assay using IgG (serology) or urea breath test (UBT).

^g^ Population source was either clinic-based or community-based.

^h^ Studies were conducted in urban, rural or both areas.

^i^ The year the H. pylori study was done.

^j^ The year H. pylori study was published.

^k^ World Health Organization region a country is situated.

**Table 2:** Descriptive statistics for characteristics of ALL incidence and H. pylori prevalence studies/countries

**ALL incidence rate (per 100,000 persons)**	**Mean**	**Median**	**s.d.**^**b**^	**Frequency**	**(%)**
Weighted ALL incidence ^a^ (28 countries)	17.61	16.40	11.75	-	-
Non-weighted ALL incidence rate (127 cancer registries)	21.68	21.25	12.62	-	-
**Characteristics of included countries (n = 28)**					
Gross National Product per capita for countries (USD$)	11375.79	9520.00	1182.10	-	-
Human Development Index for countries	0.80	0.87	0.14	-	-
Population size for countries	118137528	36416282	265588002	-	-
**Characteristics of* H. pylori *studies (n = 28)**					
H. pylori prevalence (%)	26.56	19.30	21.46	-	-
Minimum age of children (years)	2.21	1.50	2.25	-	-
Maximum age of children (years)	6.21	5.00	2.43	-	-
Interval between ALL incidence and * H. pylori * surveys (years)	4.11	3.00	4.78	-	-
Method of * H. pylori * detection					
Serological assays using IgG	-	-	-	25	89.29
Urea breath test	-	-	-	3	10.71
Population source					
Community-based	-	-	-	10	35.71
Clinic-based	-	-	-	18	64.29
Level of urbanization					
Rural	-	-	-	4	14.29
Urban	-	-	-	16	57.14
Both rural and urban	-	-	-	8	28.57
Region					
Africa	-	-	-	2	7.14
Americas	-	-	-	4	14.29
Southeast Asia	-	-	-	2	7.14
Europe	-	-	-	14	50.00
Western Pacific	-	-	-	6	21.43

^a^ Weighted ALL incidence rate for each country was obtained using the inverse variance method.

^b^ Standard deviation.

Confounders which did not have any collinearity with each other were GNP per capita (continuous) and the population source used in the *H. pylori* surveys (clinic-based or community-based samples). These were included in the further analyses.

There was a negative association between *H. pylori* prevalence and ALL incidence rates. This was non-significant when the weighted ALL incidence rates for each country were used in the model (parameter estimate = −0.10, standard error = 0.11, p-value = 0.35), However when the ALL incidence rates for all cancer registries were modelled this association became significant (parameter estimate = −0.14, standard error = 0.05, p-value = 0.01, R^2^ = 0.0485).

There was a positive association between ALL incidence rates and both confounders – the population source in the *H. pylori* surveys and the GNP per capita. This association was non-significant for the weighted ALL incidence rates (the population source used in the *H. pylori* surveys: parameter estimate = 2.98, standard error = 4.69, p-value = 0.53; GNP per capita: parameter estimate = 0.0001, standard error = 0.0002, p-value = 0.55), but became significant when the ALL incidence rates for all cancer registries were included separately (the population source used in the *H. pylori* surveys: parameter estimate = 5.40, standard error = 2.57, p-value = 0.04; GNP per capita: parameter estimate = 0.0003, standard error = 0.00009, p-value = 0.0004, R^2^ = 0.0953).

The effect of the association between the ALL incidence rates for all cancer registries and the *H. pylori* prevalence rates were attenuated (became non-significant) slightly when it was adjusted for the population source used in the *H. pylori* surveys (parameter estimate = −0.11, standard error = 0.06, p-value = 0.06). This attenuation was more pronounced when the analysis was adjusted for GNP per capita (parameter estimate = −0.05, standard error = 0.06, p-value = 0.41) and changed further when the analysis was adjusted for both the population source used in the *H. pylori* surveys and GNP per capita (parameter estimate = −0.02, standard error = 0.07, p-value = 0.82).

Generalized Estimating Equations (GEE) [14] were used to assess the relationship between *H. pylori* prevalence and ALL incidence rates among the 127 cancer registries in the 28 countries, accounting for the repeated observations within countries. The results were similar to the associations found in both the unadjusted and adjusted linear regression analyses when the ALL incidence rate for all cancer registries were modelled. For example, the unadjusted analyses for the association between ALL incidence and *H. pylori* prevalence rates had a parameter estimate of −0.14, standard error of 0.05 and p-value of 0.01 using both methods.

## Conclusion

Smith et al. [47] showed that improved hygiene, as measured by decreased prevalence of Hepatitis A virus, is associated with higher childhood ALL incidence rates. In our study we examined whether the level of sanitation, using *H. pylori* as the marker, is associated with incidence rates of childhood ALL in different countries. Our analyses demonstrated inverse association between *H. pylori* prevalence and ALL incidence rates in children. This implies that countries, where children live in better sanitary conditions and have lower *H. pylori* prevalence rates, are expected to experience increased childhood ALL incidence rates. However these associations were minor and only significant for ALL incidence rates for all cancer registries (n = 127). These associations became non-significant and smaller in magnitude, when the population source and/or the GNP per capita were added to the relationship. Furthermore, these results were unchanged when the association were examined using the Generalised Estimating Equations. Therefore, although we showed that lower prevalence of *H. pylori* and improved sanitation is associated with increased incidence of childhood ALL, our findings do not conclusively support Greaves’ [9] “ delayed infection” hypothesis.

One of the strengths in our study is the use of *H. pylori* as a biomarker for sanitation. However, as ours was an ecological study, various weaknesses are associated with the study design [14]. One is ecologic bias. This means that even though inverse associations between *H. pylori* prevalence and incidence rates of childhood ALL among populations were shown, this cannot be translated into an increased risk of incidence of ALL in an individual child known to be infected by *H. pylori*. Doing so would be erroneous and leads to ecologic fallacy. Ecologic bias can arise as a result of biases within the group studied, confounding by groups or effect modification by groups [14]. There are also more problems in ecologic studies as compared to individual-level studies with regards to the selection, control and analysis of confounders [14]. This was experienced in our study. In addition, ecologic studies do not provide assurance that disease occurrence did not precede exposure [14]. Temporality is an important criterion that needs to be met for causality in an exposure-disease relationship. Collinearity is another limitation as certain predictors, such as socio-demographic and environmental factors, tend to be highly correlated with each other than they are at individual level [14]. This is illustrated in our study, for example, between the level of urbanization and GNP per capita. Ecologic studies are also limited by lack of adequate data or if available, these data may not be comparable [14]. This was certainly seen in our study as there were insufficient data on *H. pylori* prevalence and when available, they were not comparable due to the different methodologies used in the various *H. pylori* studies. Another limitation is ALL incidence, especially in developing countries, may actually be higher since ALL may not have been diagnosed and recorded for all cases.

In view of the numerous weaknesses associated with ecologic studies, future studies looking at the association between sanitation and the incidence of childhood ALL should be done employing individual-level study designs, such as case–control studies. This will be ideal as childhood ALL is a rare disease and sanitation and *H. pylori* are not rare exposures.

## Competing interests

I declare no financial or non-financial competing interests in the submission of this manuscript.

## Author’s contribution

I performed all the steps involved in this study including the conceptualisation, design, acquisition of data, analysis and interpretation, including the drafting and submission of this manuscript.
